# Markov chain Monte Carlo Gibbs sampler approach for estimating haplotype frequencies among multiple malaria infected human blood samples

**DOI:** 10.1186/s12936-021-03841-9

**Published:** 2021-07-10

**Authors:** Gie Ken-Dror, Pankaj Sharma

**Affiliations:** Institute of Cardiovascular Research, Royal Holloway University of London (ICR2UL), London, TW20 0EX UK

**Keywords:** Haplotype reconstruction, Multiplicity of infection, Single nucleotide polymorphisms, Markov chain Monte Carlo, Gibbs sampler algorithm, Malaria

## Abstract

**Background:**

Malaria patients can have two or more haplotypes in their blood sample making it challenging to identify which haplotypes they carry. In addition, there are challenges in measuring the type and frequency of resistant haplotypes in populations. This study presents a novel statistical method Gibbs sampler algorithm to investigate this issue.

**Results:**

The performance of the algorithm is evaluated on simulated datasets consisting of patient blood samples characterized by their multiplicity of infection (MOI) and malaria genotype. The simulation used different resistance allele frequencies (RAF) at each Single Nucleotide Polymorphisms (SNPs) and different limit of detection (LoD) of the SNPs and the MOI. The Gibbs sampler algorithm presents higher accuracy among high LoD of the SNPs or the MOI, validated, and deals with missing MOI compared to previous related statistical approaches.

**Conclusions:**

The Gibbs sampler algorithm provided robust results when faced with genotyping errors caused by LoDs and functioned well even in the absence of MOI data on individual patients.

**Supplementary Information:**

The online version contains supplementary material available at 10.1186/s12936-021-03841-9.

## Background

Malaria is an infectious disease caused by protozoan parasites of the genus *Plasmodium* and transmitted to humans by *Anopheles* mosquito bites. Malaria infections in human blood often consist of several genetically distinct infections each of which is known as a clone. A “clone” consists of many individual parasites all having the same haploid genotype, which call “haplotype”. Haplotypes are a key feature in tracking anti-malarial drug resistance as genes encoding drug resistance may accumulate mutations at several codons, with each mutation increasing the level of drug resistance and possibly reducing the metabolic costs of previous mutation. Humans living in endemic areas may receive up to 1000 infective bites per-year. Polyclonal infections are common, with the number of clones within a human blood sample called the multiplicity of infection (MOI). The average number of MOI is around three in humans who live in areas of intense transmission and rarely exceeds 12 in any individual patient [1]. The presence of multiple clones each of which is haploid in a blood sample makes it difficult to identify which multiple SNPs haplotypes are present in each patient. This makes estimating the frequencies of haplotypes in the malaria population from human blood samples a challenging computational task. This method works on the principle that consider *Plasmodium* organisms in haploid state (which they are in during most their life cycle, but they are also pass-through diploid stages).

The genome of the malaria parasite *Plasmodium falciparum* was published in 2002 [2, 3], alongside that of its mosquito vector [3] and its human host (Consortium and International Human Genome Sequencing Consortium, 2001) [4], malaria genomics has led the way in the study of eukaryotic pathogens. Since then, a growing number of *Plasmodium* species genomes have been sequenced and large-scale population resequencing studies have been carried out in *P. falciparum* and several other species [2]. The development of ‘post-genomic’ tools such as SNP ‘barcoding’ panels [5, 6] has allowed parasite population structures to be studied. This approach has been used in a number of studies of *Plasmodium vivax* [2, 7]. Today, *P. vivax* is the most geographically widespread of the human malarias, and accounts for an increasing proportion of malaria cases in many endemic areas [8]. The species is more genetically diverse than *P. falciparum* [5], which may partly reflect low diversity and a more recent bottleneck in *P. falciparum*, but may also partly reflect its distinctive population structure. In co-endemic regions, the population structures of *P. vivax* have often been seen to differ from *P. falciparum*, being more genetically diverse and less geographically structured [2, 5]. The population genetics of *P. vivax* varies across its range [2].

Human blood samples can be obtained from malaria-infected patients by simple finger-prick and stored on filter paper. The dried blood spots are stable at ambient temperatures so can be easily stored and transported to a laboratory where malaria DNA can be extracted and checked for the presence of resistance mutations. Use of these surveys has been widespread [9, 10]. Traditional standard PCR-based genotyping methods still being used often underestimates MOI. Next generation sequencing technologies and genome-wide has largely increased the sensitivity in detection of polyclonal infections, but targets only partial genomic regions, which may not represent complete polymorphism in mixed infections. However, complete haplotype characterization of multiclonal infections remains a challenge due to PCR artifacts and sequencing errors and requires efficient computational tools [11].

The prevalence of mutations presence/absence in a blood sample can be directly observed. The information available for each human blood sample is an estimate of the MOI and the presence/absence of an allele at a SNP. Haplotype inference is affected by a number of sources: first, the MOI is estimated using hyper-variable genetic loci, such as *msp1*, *msp2*, *glurp* and *ta109*, which typically have an expected homozygosity of around 0.05–0.08 [12]. This may underestimate the true MOI if clones share alleles at hyper-variable loci purely by chance, or if they are low density clones missed during genotyping [13]. Second, alleles at Single Nucleotide Polymorphisms (SNPs) can only be scored as present/absent and not directly counted unless MOI equal or more than two. Third, differing assay sensitivity implication that some alleles are not detected. Different laboratories set different cut-off levels to distinguish smaller true signals from background assay noise. Some laboratories attributed signals less than 30% intensity of the main genotyping signals as ‘noise’, while other laboratories use lower cut-offs, and some rely on user subjectivity to distinguish minor peaks from technical noise. The cut-off defines as the assay’s limit of detection (LoD) [1].

These factors can have a large impact such that the haplotypes may be systematically missed [1]. The impact of detection limits must include a simulation study to know the true underlying genetic data in the simulated dataset and the observed data that would be seen in the blood samples. Several statistical approaches to estimate haplotype frequencies from multiclonal infections have been proposed including: Maximum-likelihood (ML) estimation [14], expectation–maximization (EM) algorithm [15, 16], and Metropolis–Hastings Markov chain Monte Carlo (MCMC) within a Bayesian framework [16, 17]. Wigger et al*.* [13] proposed a method for haplotype frequency estimation that uses a MCMC Gibbs sampler. Although this Gibbs sampler assumes fixed and known MOI and cannot accommodate data without MOI information. The method has not been implemented in publicly available software, and thus does not meet availability criteria for being computationally evaluated along with the other methods.

The aim of this present study is to present a novel approach MCMC Gibbs sampler for haplotype reconstruction with known or unknown MOI and to compare the results obtained to those from related statistical approaches. In addition, examined the impact of different limit of detection of the SNPs and the MOI on the results.

## Methods

The simulated datasets, estimation algorithm and statistical analysis have been implemented in the R statistical software system version 4.0.2 [18], on a 64-bit computer with 8.00 GB of random access memory and an Intel(R) Core(TM) i5-3320 M central processing unit (CPU) @ 2.60 GHz processor.

### Simulation of genotype and haplotype datasets

#### Simulation of population (haplotype) data

The simulation starts by generating a number of human blood samples, N, (1, 2,…, N) in the dataset. The multiplicity of infection (MOI) in each blood sample generated randomly by the default frequency distributions given by Jaki et al. [19] with true MOI frequencies is as follows: 1–4%, 2–40%, 3–10%, 4–10%, 5–20%, 6–5%, 7–6%, 8–5%, this reflects a distribution of MOI observed in a relative intense area of malaria transmission. Separate infections in the MOI are assumed to be genetically distinct and unrelated, haploid, asexual clones that are presumed to have been inoculated by separate mosquito bites into the same person. Each clone within the blood sample is randomly assigned an allele from each of three hyper-variable genetic markers used to estimate its MOI. The assumption is loci *msp1*, *msp2* and *ta109* whose allele frequency distributions are given by Jaki et al. [19]. Each clone is assigned a biomass randomly selected from the interval 10^9^–10^11^, the biomass is the total number of parasites in the human and this sampling interval is typical for symptomatic malaria infections. The relative biomass of each clone (i.e., its proportion of the total biomass) is calculated as that clone’s biomass divided by the total biomass in the patient. The genotyping signal from a SNP or MOI allele will be assumed to be proportional to the relative biomass of parasites containing that allele. This approach was used to generate genetic datasets, assumed: 100 blood samples per-dataset, diallelic SNPs either resistant or sensitive resistance allele frequencies (RAF) at each codon ranging from 1%, to 50%, and linkage equilibrium (LE) between all SNPs and MOI markers. 1000 datasets were generated and analysed assuming differing LoD: 0.0/0.0, 0.1/0.05, 0.2/0.1, 0.3/0.15 where the first number is LoD_SNP_ and the second is LoD_MOI_.

#### Simulation of observed (genotype) data

Genotypes are the observable data obtained on human blood samples and are subjected to the sources of genetic ambiguity, genotyping errors arising from LoD and the fact that different combination of haplotypes may give rise to the same observed genotype. The true genetic data are, therefore, processed as follows to simulate what would be observed in the blood samples.

##### Observed MOI

The strength of each genotyping signal is calculated from their relative biomasses. The cut-off for distinguishing true signals from ‘noise’ may differ slightly from that used for SNPs, which is why having different detection limits for LoD_SNP_ and LoD_MOI_. The novel algorithm assumes a signal less than a certain proportion of the major signal, this proportion being denoted LoD_MOI_, is regarded as noise. If LoD_MOI_ = 0.1, signals < 10% of the maximum would be regarded as noise and would not contribute to the observed blood sample genotype. The observed MOI is then calculated as the maximum number of the alleles observed at three hyper-variable genetic markers *msp1*, *msp2* and *ta109*.

##### Observed genotypes

These are calculated in an analogous manner to MOI, by assuming that a clone’s biomass determines its contribution to the genotyping signal. The total signal for each allele at each SNP is then calculated and compared to the defined LoD_SNP_ to find which alleles are detectable and contribute to the observed blood genotype (Additional file 1: Table S1).

Finally, a reality check was run on the simulated blood dataset, search for samples with observed MOI = 1 and one of the SNPs is heterozygous. These observations are incompatible and generally occur when MOI ≥ 2 but appears to have MOI = 1 for one of two main reasons. Firstly, the ≥ 2 clones are identical at all three MOI loci purely by chance such that the observed MOI = 1. Secondly, the clones do differ at one or more MOI loci but a difference in genotyping sensitivity (LoD) between MOI and SNPs means only a single MOI allele is detected at each hypervariable locus, but a heterozygote is detected at one of the SNPs. In both cases, the MOI is reset to have a value of 2 as would likely occur when processing clinical samples.

### Novel haplotype reconstruction methods

#### The Markov chain Monte Carlo (MCMC) Gibbs sampler

The Gibbs sampler also known as the Glauber dynamics or the heat-bath algorithm, is a leading MCMC method for obtaining a sequence of observations which are approximated from a specified multivariate probability distribution, when direct sampling is difficult [20]. The Gibbs sampling algorithm generates a new sample from the distribution of each variable based upon the conditional distribution among the current values of the other variable [20–22]. The Gibbs sampler is a popular MCMC algorithm and is widely used in phylogenetic analysis, sequence motif discovery and haplotype estimation. The algorithm consists of several steps and is explained in detail in the Additional file 1.

#### Existing statistical methods of haplotype reconstruction

There are five other published methods that are available to use: MalHaploFreq software, Maximum likelihood (ML) estimation using a hill climbing algorithm [14] (hereon called “MHF”). Expectation–maximization (EM) algorithm malaria.em [15] estimation using efficient iterative maximum likelihood approach (hereon called “R-EM”. A Bayesian approach [17] estimation using a Metropolis–Hastings Markov chain Monte Carlo (hereon called “Bayesian”). Another expectation–maximization (EM) algorithm [16] estimation using maximum likelihood approach that incorporates MOI (hereon called “EM”). And the Markov chain Monte Carlo (MCMC)-algorithm [16] using acceptance/rejection algorithm (hereon called “MCMC”). Summary of the statistical methods for haplotype reconstruction presents in the Additional file 1: Table S1.1.

### Evaluation of different statistical methods

Thousand datasets were simulated assuming that resistance is encoded at three loci there are eight resistance haplotypes. Each dataset is obtained by a process of five sequential steps:

I. The population frequencies of haplotypes are defined by selecting a RAF for each locus at random and the 8 population haplotype frequencies obtained assuming linkage equilibrium between the alleles;
II. A field survey of malaria blood samples is simulated. Each patient in the dataset has an MOI assigned at random according to the frequencies given above. The malaria clones a number equal to the MOI are then sampled at random according to the true population frequencies of resistance haplotypes and polymorphic markers (*msp1*, *msp2*, *ta109*). The sampled resistance haplotype frequencies in the dataset will differ from the true frequency due to this sampling process;
III. The MOI polymorphic markers and resistance SNPs in each patient are then processed to obtain the genotypes observed in the blood samples taken from patients depending on the LoD;
IV. The estimated resistance haplotype frequencies are obtained from each of the statistical programmes;
V. The estimated haplotype frequency from dataset is used to evaluate the performance of the six methods. Each of these five steps is repeated for each of the 1000 datasets. The datasets and selected haplotype in each dataset are kept the same for each of the six-analysis method, this allows a direct comparison between the different methodologies used to infer haplotype frequencies.

The performance and the accuracy of the different methods is measured as follows: ‘*P*’ is a vector whose number of elements equal *h,* the number of potential haplotypes in the malaria population in the sample case 3 resistance SNPs (*h* = 2^3^ = 8). The elements of the vector are indicated by the superscript *i*. The population and sampled values are compared with the estimated value as:

I. The correlation coefficient (*R*^*2*^) between population or sample and estimated haplotype frequency;
II. Similarity index (*I*_*F*_) [23, 24] to examine how close the computationally estimated haplotype frequencies are to the population or sampled haplotype frequencies as: $${I}_{F=}{\sum }_{i=1}^{h}{\mathrm{min}}({Pi}_{estimated,}{Pi}_{population or sample})=1-1/2{\sum }_{i=1}^{h}|{Pi}_{estimated}-{Pi}_{population or sample}|$$. This measure incorporates all *h* haplotype frequencies and thus captures the overall difference between estimated and population or sample frequencies. It varies between one, when population or sample and estimated haplotype frequencies are identical, and zero when estimated haplotypes frequencies tending to zero;
III. The mean squared error (*MSE*) [25, 26] was calculated as: $$MSE=\left[{{\sum_{i=1}^{h}}(Pi_{estimated}-{Pi}_{population or sample})^{2}}\right]/h$$ the estimated and the population or sample haplotype frequency of *i* haplotype, *h* is the number of haplotype frequencies in the population;
IV. Change coefficient *C* [27, 28] assess the scaled change in haplotype frequencies and was calculated as: $${C}_{i}=(|Pi_{estimated}-{Pi}_{population or sample}|)/Max[(Pi_{estimated},\,{Pi}_{population or sample}]$$. The coefficients were computed for each possible haplotype across statistical methods and presented as difference of estimation (%). The value of the coefficient *C* ranges from 1 to − 1, the value 0 indicating that the haplotype frequency estimated, and the haplotype frequency population or sample are identical. Positive values indicate that haplotype frequency estimates tend to be larger than the population or sample frequency;
V. The validity of the methods measures how often the population and sampled frequencies fall within the 95% CI of the estimated frequency. In addition, the speed of the analyses which is self-explanatory recorded.

## Results

The estimated haplotype frequencies with simulated population and sample haplotype frequencies across six statistical methods MHF (MalHaploFreq), R-EM (malaria EM), Bayesian (Bayesian statistic), EM (EM-algorithm), MCMC (Markov chain Monte Carlo), Gibbs (Gibbs sampler) and four conditions of LoD_(SNP/MOI)_ showed high concordance. Table 1 shows the absolute deviation of the estimated haplotype frequencies from population and sample haplotype frequencies. The correlation coefficient (*R*^*2*^) is slightly higher by 0.50–1.30% in the sample haplotype compared to the population haplotype among all statistical methods. Increasing both LoD_MOI_ and LoD_SNP_ decreases the correlation coefficient by 0.10–0.30% among EM method. Conversely, increasing both LoD_MOI_ and LoD_SNP_ increased the correlation coefficient by 0.30–4.90% among MHF, R-EM, Bayesian, MCMC and Gibbs methods. The difference between correlation coefficients among statistical methods is less than 6.4% among population haplotype frequencies and 5.7% among sample haplotype frequencies. The bias is slight and changes with limits of detection.

**Table 1:** The correlation (*R*^*2*^) of the estimated haplotype frequencies with simulated population and sample haplotype frequencies across statistical methods and four conditions of LoD_SNP_ (30%, 20%, 10%, 0%) and LoD_MOI_ (15%, 10%, 5%, 0%)

	MHF	R-EM	Bayesian	EM	MCMC	Gibbs
Population haplotype (LoD_SNP_/LoD_MOI_)						
0/0	0.949	0.913	0.955	0.977	0.973	0.961
0.10/0.05	0.953	0.932	0.960	0.978	0.980	0.970
0.20/0.10	0.962	0.949	0.960	0.977	0.981	0.975
0.30/0.15	0.957	0.962	0.960	0.974	0.976	0.977
Sample haplotype (LoD_SNP_/LoD_MOI_)						
0/0	0.960	0.926	0.962	0.983	0.978	0.966
0.10/0.05	0.963	0.944	0.968	0.985	0.986	0.976
0.20/0.10	0.971	0.960	0.968	0.984	0.987	0.982
0.30/0.15	0.967	0.972	0.967	0.982	0.983	0.984

Higher value represents higher accuracy

*MHF* MalHaploFreq, *R-EM* malaria em, *Bayesian* Bayesian statistic, *EM* EM algorithm, *MCMC* Markov chain Monte Carlo, *Gibbs* Gibbs sampler, *LoD* limit of detection, *SNP* single nucleotide polymorphisms, *MOI* multiplicity of infection

Table 2 shows the similarity index (*I*_*F*_) of the estimated haplotype frequencies compared population and sample haplotype frequencies. The six statistical methods provided similarity index (*I*_*F*_) values very close to each other. The similarity index is higher by 0.02–1.2% in the sample haplotype compared to the population haplotype among all statistical methods. The difference between similarity indexes among statistical methods is less than 6% among population and sample haplotype frequencies. Increasing both the LoD_SNP_ and LoD_MOI_ decreases the similarity index between 0.4–2% in MHF, Bayesian, and EM methods. Conversely, R-EM, MCMC and Gibbs methods shows increasing values of *I*_*F*_ between 1 to 4%. The increased *I*_*F*_ and *MSE* in three tested methods and decreasing in the other three tested methods look like random patterns. The changes are small, not statistically significant and for some of the methods the increases and decreases are not monotonous, but rather, the *I*_*F*_ and the *MSE* jump around between higher and lower values without an obvious pattern. This tendency is reflected in the mean squared error (*MSE*) statistics. Table 3 shows the *MSE* of the estimated haplotype frequencies around the population and sample haplotype frequencies. The *MSE* is lower by 0.005–0.027 in the sample haplotype compared to the population haplotype among all statistical methods. The difference between *MSE* between statistical methods is less than 0.128 among population haplotype frequencies and 0.117 among sample haplotype frequencies. Increasing both the LoD_SNP_ and LoD_MOI_ increased the *MSE* between 0.012 and 0.074 among MHF, Bayesian, and EM methods. Conversely R-EM, MCMC and Gibbs methods decrease the *MSE* values between 0.009 and 0.095.

**Table 2:** The similarity index (*I*_*F*_) of the estimated haplotype frequencies with simulated population and sample haplotype frequencies across statistical methods and four conditions of LoD_SNP_ (30%, 20%, 10%, 0%) and LoD_MOI_ (15%, 10%, 5%, 0%)

	MHF	R-EM	Bayesian	EM	MCMC	Gibbs
Population haplotype (LoD_SNP_/LoD_MOI_)						
0/0	0.906	0.879	0.910	0.938	0.915	0.919
0.10/0.05	0.911	0.894	0.913	0.943	0.938	0.934
0.20/0.10	0.905	0.909	0.909	0.940	0.945	0.940
0.30/0.15	0.889	0.918	0.898	0.930	0.930	0.932
Sample haplotype (LoD_SNP_/LoD_MOI_)						
0/0	0.917	0.886	0.917	0.942	0.917	0.921
0.10/0.05	0.923	0.904	0.922	0.950	0.942	0.939
0.20/0.10	0.915	0.920	0.917	0.948	0.953	0.948
0.30/0.15	0.897	0.930	0.906	0.938	0.937	0.942

Higher value represents higher accuracy

*MHF* MalHaploFreq, *R-EM* malaria em, *Bayesian* Bayesian statistic, *EM* EM algorithm, *MCMC* Markov chain Monte Carlo, *Gibbs* Gibbs sampler, *LoD* limit of detection, *SNP* single nucleotide polymorphisms, *MOI* multiplicity of infection

**Table 3:** The mean squared error (*MSE*) of the estimated haplotype frequencies with simulated population and sample haplotype frequencies across statistical methods and four conditions of LoD_SNP_ (30%, 20%, 10%, 0%) and LoD_MOI_ (15%, 10%, 5%, 0%)

	MHF	R-EM	Bayesian	EM	MCMC	Gibbs
Population haplotype (LoD_SNP_/LoD_MOI_)						
0/0	0.103	0.178	0.091	0.050	0.085	0.097
0.10/0.05	0.108	0.138	0.088	0.043	0.047	0.065
0.20/0.10	0.120	0.103	0.108	0.051	0.040	0.050
0.30/0.15	0.177	0.086	0.141	0.072	0.076	0.062
Sample haplotype (LoD_SNP_/LoD_MOI_)						
0/0	0.082	0.158	0.077	0.041	0.080	0.091
0.10/0.05	0.086	0.117	0.072	0.032	0.038	0.055
0.20/0.10	0.095	0.081	0.089	0.036	0.027	0.037
0.30/0.15	0.150	0.063	0.122	0.053	0.058	0.044

Lower value represents higher accuracy

*MHF* MalHaploFreq, *R-EM* malaria em, *Bayesian* Bayesian statistic, *EM* EM algorithm, *MCMC* Markov chain Monte Carlo, *Gibbs* Gibbs sampler, *LoD* limit of detection, *SNP* single nucleotide polymorphisms, *MOI* multiplicity of infection

Table 4 shows the average change coefficient *C* of the estimated haplotype frequencies compared population and sample haplotype frequencies for haplotype frequency > 5%. The change coefficient *C* is lower by 0.5–2.4% in the sample haplotype compared to the population haplotype among all statistical methods. The difference between change coefficient *C* among statistical methods is less than 12% among population haplotype frequencies and 11.7% among sample haplotype frequencies. Increasing both the LoD_SNP_ and LoD_MOI_ increases the change coefficient *C* between 1.0–1.8% in MHF, Bayesian, and EM methods. Conversely, R-EM, MCMC and Gibbs methods shows decreasing values of change coefficient *C* between 1.7% and 8.3%. There was a tendency for the estimates to cluster more closely to the population and sample haplotype frequencies at high frequencies, showing that there is a tendency for high-frequency haplotypes to be more accurately estimated among all statistical methods.

**Table 4:** The average change coefficient (*C*) of the estimated haplotype frequencies with simulated population and sample haplotype frequencies for haplotype frequency > 5% across statistical methods and four conditions of LoD_SNP_ (30%, 20%, 10%, 0%) and LoD_MOI_ (15%, 10%, 5%, 0%)

	MHF	R-EM	Bayesian	EM	MCMC	Gibbs
Population haplotype (LoD_SNP_/LoD_MOI_)						
0/0	20.8	25.2	18.7	13.2	17.7	16.1
0.10/0.05	19.3	22.3	18.0	12.4	13.4	13.7
0.20/0.10	19.8	19.7	18.6	13.0	11.9	12.9
0.30/0.15	22.0	17.8	20.1	14.6	14.4	14.4
Sample haplotype (LoD_SNP_/LoD_MOI_)						
0/0	18.8	23.7	17.4	12.0	17.2	15.4
0.10/0.05	17.0	20.5	16.3	10.9	12.4	12.5
0.20/0.10	17.7	17.5	17.1	11.4	10.4	11.3
0.30/0.15	20.6	15.4	18.8	13.0	13.1	12.8

Lower value represents higher accuracy

*MHF* MalHaploFreq, *R-EM* malaria em, *Bayesian* Bayesian statistic, *EM* EM algorithm, *MCMC* Markov chain Monte Carlo, *Gibbs* Gibbs sampler, *LoD* limit of detection, *SNP* single nucleotide polymorphisms, *MOI* multiplicity of infection

Table 5 shows the validity of the methods quantifies as how often the population and sample haplotype frequencies fall out of the 95% confidence intervals. The percentage of results falling outside of the 95% CI is slightly lower, by 0.1–4%, in the sample haplotype frequencies compared to the population haplotype frequencies among all statistical methods. The difference between percentage of results falling outside of the 95% CI among statistical methods is less than 13.9% among population haplotype frequencies and 10.1% among sample haplotype frequencies. Increasing both the LoD_SNP_ and LoD_MOI_ increased the error rates produced by Bayesian and MHF approaches with 15.1–13.4%, and 8.7–6.8% of estimates lying outside the 95% CI. However, the R-EM, EM, MCMC and Gibbs methods shows small changes in LoD with variation in the percentage falling outside the 95% CI between 0.1–3.9% across the four LoD assumptions.

**Table 5:** Percentages of simulated sample haplotype frequencies and population haplotype frequencies that fall outside the confidence intervals of the estimated haplotype frequencies across statistical methods and four conditions of LoD_SNP_ (30%, 20%, 10%, 0%) and LoD_MOI_ (15%, 10%, 5%, 0%)

	MHF	R-EM	Bayesian	EM	MCMC	Gibbs
Population haplotype (LoD_SNP_/LoD_MOI_)						
0/0	4.90	5.90	14.60	0.70	4.00	3.00
0.10/0.05	6.20	5.70	15.10	1.00	1.30	1.90
0.20/0.10	11.40	5.90	18.10	2.00	1.80	2.10
0.30/0.15	20.00	8.10	23.30	4.30	4.40	3.80
Sample haplotype (LoD_SNP_/LoD_MOI_)						
0/0	6.90	8.30	14.80	4.50	8.00	6.40
0.10/0.05	7.50	7.50	14.50	4.40	5.10	4.80
0.20/0.10	11.50	6.80	17.10	4.40	4.40	4.60
0.30/0.15	20.30	8.20	21.60	5.40	5.40	5.20

Lower value represents higher accuracy

*MHF* MalHaploFreq, *R-EM* malaria em, *Bayesian* Bayesian statistic, *EM* EM algorithm, *MCMC* Markov chain Monte Carlo, *Gibbs* Gibbs sampler, *LoD* limit of detection, *SNP* single nucleotide polymorphisms, *MOI* multiplicity of infection

Table 6 shows the computational time for the statistical methods. There is a big difference between the statistical methods of almost 517 s. Increasing LoD_MOI_ and LoD_SNP_ decreased the time of the analysis by 79% among MHF, 25% among R-EM, 89% among Bayesian, 4% among EM, 82% among MCMC and 34% among Gibbs.

**Table 6:** The computational time (seconds) of the estimated haplotype frequencies across statistical methods and four conditions of LoD_SNP_ (30%, 20%, 10%, 0%) and LoD_MOI_ (15%, 10%, 5%, 0%)

	MHF	R-EM	Bayesian	EM	MCMC	Gibbs
(LoD_SNP_/LoD_MOI_)						
0/0	29.8	517.5	3.5	19.9	5.4	25.5
0.10/0.05	31.1	353.6	3.4	1.9	5.2	19.2
0.20/0.10	25.6	227.5	3.3	1.3	4.8	13.1
0.30/0.15	23.4	127.7	3.1	0.9	4.4	8.7

Lower value represents faster calculation

*MHF* MalHaploFreq, *R-EM* malaria em, *Bayesian* Bayesian statistic, *EM* EM algorithm, *MCMC* Markov chain Monte Carlo, *Gibbs* Gibbs sampler, *LoD* limit of detection, *SNP* single nucleotide polymorphisms, *MOI* multiplicity of infection

Additional file 1: Tables S2–S6 shows the performance of the statistical methods when MOI is unknown. In addition, Additional file 1: Tables S7–S11 shows examples of haplotype defined at 2 SNPs. Similar patterns were observed when haplotypes were defined at less than 3 SNPs.

## Discussion

This study proposed the Gibbs sampler statistical method for haplotype reconstruction among multiple malaria infected human blood samples. These methods present a greater accuracy with high limits of detection of the SNPs or the MOI, robust validity performance, deals with missing MOI and the ability to return the probabilities of possible haplotype combination in each individual and the uncertainly probability of the haplotype frequencies.

It was considered important to recognize the technical limitations of genotyping so three values for levels of detection (LoDSNP and LoDMOI) were investigated. There are differences between the statistical methods especially with increasing LoD_MOI_ and LoD_SNP_, the true MOI is under-estimated in around 5% of patients even if the LoD_MOI_ are zero [16]. The MHF method is accurate and valid when the LoD_MOI_ and LoD_SNP_ are zero, but increased LoD dramatically decreased the accuracy and the validity of the result. The R-EM method presents the opposite, its accuracy and the validity increase when the LoD_SNP_ and LoD_MOI_ increases. The Bayesian method appears exhibits accurate and valid when the LoD_SNP_ and LoD_MOI_ are zero but increasing the LoDs dramatically decreases the accuracy and the validity of the results. The EM method obtains highly accurate and valid results irrespective of the LoD_SNP_ and LoD_MOI_ values. The MCMC and Gibbs method obtains results that are sensitive to LoD levels, its accuracy and validity both increase as the LoDSNP and LoDMOI increase. All the methods suffer from dimensionality, but the novel method still gave better results than the existing methods over all combinations of different limits of detection (LoD) of the SNPs and the MOI 0.0/0.0, 0.1/0.05, 0.2/0.1, 0.3/0.15, respectively.

Differences in the sources of data and assumptions for different methods can arise for several reasons. Firstly, the assumption that blood samples are representative of the population and incorporation of patient-level MOI estimates, or not use this assumption and regard the MOI as a fixed quantity. Second, assumptions about independence of different loci in the same infections (i.e., linkage disequilibrium) or about independence of the co-infecting clones and linkage disequilibrium. Third, assumptions about detectability of different clones, whether and how this varies, and whether dominant clones affect the detection of minor clones. Finally, assumptions about the resolution of the typing system and its ability to resolve genetically related clones arising from distinct inoculations or from inoculation of sibling clones arising from the same meiosis in the mosquito. The statistical methods are algorithms for optimization, iterative, starting with an initial value that belongs to the parameter space. At each step of the algorithms a new parameter value is chosen, hopefully a value closer to the terminal target value than the previous one. The algorithms are stopped when it converges, namely when the new value is very close to the current value. The value of the parameter that was selected when the algorithm converged is declared to be the maximiser.

There are differences between the statistical methods validity performance because the differences way the CI was calculated. The MHF method calculate 95% CI boundaries as occurring when the likelihood is less than 2 log units below the maximum-likelihood. The R-EM method calculate 95% CI from the standard error of the estimated haplotype frequencies. The Bayesian methods calculate 95% CI as quintiles from haplotype frequency matrix. The EM, MCMC and Gibbs statistical methods calculate 95% CI base on exact binomial tail areas. When LoD is zero the EM, MCMC and Gibbs methods produce very narrow CI, while MHF is about correct containing 95% of the values while the Bayesian method produces CI that are too wide with only about 85% of true values being contained within the CI. The MHF and Bayesian methods produced haplotype estimates that lay 25% outside the CI when molecular detection increases.

To estimate the haplotype frequency, the Gibbs sampler methods used two required inputs, genotypes, and MOI, or one if the MOI was missing. The Gibbs sampler draws iteratively from conditional distributions particularly useful and lower in dimension rather than drawing directly from the joint distribution with which it may not be always easy to work. While the Gibbs sampler relies on conditional distributions, the Bayesian and MCMC methods bases on Metropolis–Hastings sampler uses a full joint density distribution to generate a candidate draws. The candidate draws are not automatically added to the chain but rather an acceptance probability distribution is used to accept or reject candidate draws. These methods are sensitive to the step size between draws. Either too large or too small of a step size can have a negative impact on convergence. The Gibbs algorithm sampling likely haplotypes for all subjects does not need to consider every possible haplotype unlike the EM-algorithm which must sum over every possible haplotype during the E-step. This property of the Gibbs sampler makes it better suited to deal with situations where there are many possible haplotypes, many markers, and MOI. While the EM-algorithm will converge to a maximum, it may be only a local maximum. However, the Gibbs sampler may get trapped in a local mode, but it does have a chance of escaping such a mode and finding the true regions of parameter space with high probability.

The convergence diagnostics plots present in the Additional file 1: Figures S1–S6 demonstrates clear convergence. The algorithm successfully converged to reach a stationary distribution after a few runs. The chain did not get stuck in certain areas of the parameter space, indicating poor mixing. The median of the shrink factor does not increase above 1.1 among all haplotype frequency groups. The Gelman and Rubin's convergence diagnostic the scale reduction factors for each parameter is 1.09 maximum value at each parameter for 500 iterations and it decreases to 1.05 maximum value at each parameter for 1000 iterations. A factor of 1 implies that between and within chain variances are equal, larger values suggest that there is still a notable difference between chains. Shrink values below 1.1 or 1.05 acceptable for practical purposes [29]. Gelman and Rubin [30] and Brooks and Gelman [31] suggest that the maximum Gelman–Rubin diagnostic across all model parameters values greater than 1.2 for any of the model parameters should indicate non-convergence. In addition, the mean plots present how well the chains are mixing and how the two chains go in the same direction. More iteration cause further decreases in the scale reduction factor however, Raftery and Lewis [32, 33] test the number of iterations and suggest a minimum of 300 iterations. These diagnostics tend to be conservative so that more iterations may be necessary. Heidelberg and Welch diagnostic [34, 35] calculates a test statistic to change the null hypothesis that the Markov chain is from a stationary distribution, the chain passes the test, so the chain does not need to run longer. In addition, the chain passes the Geweke diagnostic test [36] that takes two nonoverlapping parts the first 0.1 and last 0.5 proportions of the Markov chain and compares the means of both parts, using a difference of means test to see if the two parts of the chain are from the same distribution (null hypothesis). Accordingly, the current algorithm runs 500 iterations and 200 burn ins. The algorithm allows the user to choose the number of iterations, number of chains and number of burn in.

The Gibbs sampler methods examines possible haplotype combination in an individual that could plausibly give rise to the observed genotype and obtain the probability that any given patient harbours a drug resistant haplotype. The presence of a putative resistant haplotype can be inferred in individual patients and the probability of their presence used as a weighting in a logistic regression predicting the therapeutic outcome (cure/fail) of drug treatment. A positive impact of the putative-resistant haplotype on therapeutic outcome would indicate it truly affects resistance levels.

The R-EM, Bayesian, EM, MCMC and Gibbs can calculate the haplotype frequencies when MOI information on a patient was unmeasured or missing. Every one of the methods makes a prior assumption on the probability distribution on the number of infections per-individual. The Gibbs method (Additional file 1: Tables S2–S6) obtain higher values among correlation (*R*^*2*^) and similarity index (*IF*) in addition, to lower values among *MSE*, average change coefficient (*C*), and haplotype frequencies that fall outside the CIs that represents more accurate frequency estimates. Furthermore, the Gibbs method was slightly less affected by LoD of the SNPs and the MOI than compared to the related statistical approaches. The haplotype frequencies estimate shows excellent correlation with the population or sample values even in the absence of MOI information among the Gibbs method.

While all the methods have similar accuracy, they differ in important aspects. The MHF method has a faster runtime for analyses but is limited to analysing up to three SNPs and cannot deal with missing values. The R-EM method can deal with missing values, analyse more than three SNPs but required a considerable amount of computational time. The Bayesian method base on prior distribution has a fast runtime, but limited to handling up to seven SNPs, although it can deal with missing values. The EM method is fast, can deal with missing values, and analyse more than three SNPs. The MCMC method is fast, can deal with missing values, and analyse more than three SNPs. The Gibbs method is fast, can deal with missing values, is unlimited by the number of SNPs analysed but calculating the frequencies of haplotypes that are defined at a large number of SNPs increases the computational time, the magnitude of this increase depending on the computer memory (Additional file 1: Table S1.1).

The simulations were limited to two and three SNPs to simplify the comparison. Results from haplotypes defined at two SNPs are presented in Additional file 1: Table S7–S11, the same pattern irrespective of whether haplotypes are defined at two or three loci. The examples were limited to three SNPs because the complexity of calculations rises exponentially with the number of SNPs and it is rarely necessary in practice to analyse more than three SNPs simultaneously [14] when investigating drug resistance haplotypes. However, calculating large number of SNPs increases the calculation time and depends on available computer memory. The reduction in time taken to run the analyses is that as LoD increases, the observed MOI and genetic diversity within patients tends to decrease, consequently the datasets become slightly simpler and their analysis faster. These statistical methods work on Bi-allelic SNPs and are not created to deal with multiallelic SNPs. The Gibbs method can be extended to deal with multiallelic SNPs, like most algorithms for optimization this situation will increases the parameter space, the iterative process, and the computational time. Multiallelic SNPs are not observed very frequently (< 2%) but often taken as a sign of a noisy region where artifacts are likely, unless looked at in very large cohorts [37, 38]. It is requested very high number of genes under multiallelic balancing to explain any genetic pattern, and this high amount has never been seen in the literature. The Gibbs sampling method presented in this work is only applied to simulated data and has not been tried on real data.

## Conclusion

It is shown that the Gibbs method proposed has advantages over previous methods of inferring haplotype frequencies. It is robust to chance misclassification of MOI and to genotyping detection limits if MOI information is absent. The Gibbs method converges on accurate estimates of haplotype frequencies irrespective of initial assumptions of haplotype and MOI frequencies. The R code used for these simulations and analyses are freely available on request to GKD.

## Supplementary Information

**Additional file 1:** Includes data that support and expand some of the interpretations and conclusions drawn in the main text.

## Data Availability

Field data will not be shared. We are prepared to share compute code and our simulated datasets on request.

## Abbreviations

EM: Expectation–maximization
MCMC: Markov chain Monte Carlo
MOI: Multiplicity of infection
RAF: Resistance allele frequencies
LoD: Limit of detection
MHF: MalHapFreq
R-EM: Malaria.em
ML: Maximum likelihood
LE: Linkage equilibrium
SNPs: Single nucleotide polymorphisms
*I*_*F*_: Similarity index
MSE: Mean squared error
CI: Confidence intervals
