# Supplementary material for: Markov chain Monte Carlo Gibbs sampler approach for estimating haplotype frequencies among multiple malaria infected human blood samples
Source: Malar J. 2021 Jul 10;20:311. doi: 10.1186/s12936-021-03841-9 (PMC8272262; doi:10.1186/s12936-021-03841-9)

Additional file 1:

Supplementary

This Additional file includes data that support and expand some of the interpretations and conclusions drawn in the main text, but whose inclusion would detract from the main argument.

Table S1: How malaria datasets are simulated. The 'population' frequencies of different MOI classes, polymorphic markers (*msp1*, *msp2*, *ta109*) and resistance haplotypes in the local malaria population are first defined. A number of patients are then simulated, 5 in this case but more usually 100. For each patient a MOI is first sampled according to the local "population" frequencies (which will depend on local transmission intensity). This MOI then determines the number of malaria clones in the patient. These clones are then simulated. The first step is to assign a biomass to the clone. The clone polymorphic markers are assigned at random according to the local true frequencies. Finally, a resistance haplotype is assigned to the clone, again sampled from the local true frequencies. This process is repeated for each clone in each patient and gives rise to the data given in black font below. The genetic signal observed in each patient (last two columns) is then calculated as described in the main text. In this example, genetic signals are not detected if they constitute ≤10% of the biomass (f.BIOMASS gives relative biomass for each clone in a patient) so the signals stuck-through in the Table are not actually observed. What is actually observed, and available for analysis, is the information given in red; genotyping limits produce errors, and those erroneous data are indicated by a matrix: they are the data available to the researcher but do not truly reflect the genetic data of the parasites in that patient.

Patient #	MOI	BIOMASS	f.BIOMASS	*msp1*	*msp2*	*ta109*	Haplotype	Observd MOI	Observed genotype
1	1	5.29E+10	1.000	10	34	3	112	1	112
2	3	8.06E+09	0.100	24	23	5	112	1*	111*
		6.48E+10	0.803	20	6	5	111		
		7.86E+09	0.097	16	27	5	112		
3	2	5.06E+10	0.474	24	35	3	111	2	111
		5.62E+10	0.526	1	34	4	111		
4	2	5.52E+10	0.487	21	34	4	122	2	133
		5.81E+10	0.513	18	33	4	111		
5	3	3.16E+10	0.432	23	32	9	111	2*	133*
		1.35E+09	0.018	21	28	7	112		
		4.03E+10	0.550	23	27	9	122		

Haplotype is the resistance haplotype for each clone. It is defined at 3 SNPs, for each clone: 1=wildtype, 2=mutat.
Observed genotype is observed genotype for each patient. It is defined at three SNPs; for each SNP: 1=wildtype alone, 2=mutant alone, 3=both wildtype and mutant genetic signals observed in the blood sample.

The Gibbs sampler algorithm

The observed data consists of MOI and SNP genotypes (wildtype, resistant, mixed) at several genetic loci. Let n represent the number of blood sample (sample size), i is an index used to refer to an individual blood sample (i=1, …, n), j is an index used to refer to a unique haplotype combination within a blood sample i, $h_{(i,j)}$ is a set of haplotypes, s is the number of SNPs genotyped, q is the max MOI identified in the dataset, z is the number of potential haplotypes in the population (2^s), m is a vector of MOIs for each patient, $m = (m_1, ... m_n)$. G is a vector of genotype group for each patient, $G = (g_1, ... g_n)$. H is a vector of haplotype sets, $H = (H_1, ... H^z)$ and θ is a vector of estimated haplotype frequencies, $\theta = (\theta_1, ... \theta^z)$. The G_i is the number of patients with genotype group i, $\Sigma_{i \in Gi}$ is summation of all individuals i that are in genotype group G_i.

The algorithm begins by assigning a sequence of haplotypes from a multinomial distribution (initial guess) that can give rise to the observed SNP genotype in each patient. These haplotypes are held in a matrix D whose size depends on the sample size (n, rows), and the maximum number of observed MOI (q, columns). The elements of this matrix are the haplotypes that are consistent with the observed patient genotype. The elements of matrix D can then be used to obtain the current estimates of haplotype frequencies by calculating haplotype proportions which are, stored in vector θ. The next step is to choose an individual at random from among those individuals with ambiguous genotypes (individuals where more than one sequence of haplotypes can give raise to the observed genotype). An update is proposed by simulating a new sequence of haplotypes consistent with observed genotype and MOI base on the conditional distribution. Determine the conditional distributions for each individual depends on the following metric:

$$\Upsilon = log\left(\prod_{i=1}(\theta_{(h_{i,j})}) \Big/ \sum_{i=1} \prod_{i=1}(\theta_{(h_{i,j})}) + 1 \right) * \sum_{i=1} \left(\sum_i H_i! \Big/ m_i! * (\sum_i H_i - m_i)! \right) * \sum_{i \in} G_i \qquad \text{(S1)}$$

The update is always accepted, the ϒ is the probability of accepting the update. At the end of each iteration the current estimate of haplotype frequencies in θ is updated. This process of updates until the estimated haplotype frequencies converged stationary distribution. Iterations defined as being completed when every heterozygous individual has been selected and tested for an update. Patients are selected at random (there is no set sequence) but every patient can only be selected one time in each iteration. The algorithm makes at least 500 iteration and the trace and autocorrelation output as graphs (supplementary Figures S1 to S6). The algorithm tested with run 500 iteration simulates multiple chains with different starting values and reached the same results. The trace plots the iteration number against the sampled values (theta) for each variable (haplotype) in the chain, with a separate plot per variable (haplotype). It can show when the chain gets stuck in certain areas of the parameter space, which indicates bad mixing. The autocorrelation is the correlation between the theta values in the current iteration with their values in the previous iteration. This enables the user to check that convergence to a stationary distribution has occurred, to identify the burn in period, and discount frequency estimates made during this burn-in period. This can be done by visual inspection of the graphs.

When MOI information on a patient was unmeasured or missing, the algorithm proceeds as follows. To initialise the analyses each induvial is assigned an MOI according to the distribution frequency used by Jaki et al. [7] and all haplotype combinations within this MOI that give rise to the observed genotype are listed and processed. If no haplotype combinations can generate the observed genotype then another MOI is assigned, again using the MOI distribution of Jaki et al. [7]. The Gibbs sampler then proceeds as described above. An alternative algorithm would be to list all the MOI and their possible haplotype combinations for a patient (and scaling by the probability of that MOI in the population). Initial analyses

using this approach incurred a very substantial speed penalty for a very small improvement in accuracy, hence out approach above that only a single MOI is investigated for each patient.

The confidence interval around haplotype frequency estimates

Once haplotype frequencies have been estimated, by the Gibbs method, the confidence interval (CI) around these estimates are calculated from the exact binomial tail areas [19] that are usually considered as the gold standard. The lower and upper bound of the interval are defined via quantiles of the F distribution: ${}^{x}\big/_{x+(n-x+1)F^{2n-2x+2}_{2x,1-\alpha/2}} \leq \theta_i \leq {}^{(x+1)F^{2x+2}_{2n-2x,1-\alpha/2}}\big/_{x+(n-x+1)F^{2x+2}_{2n-2x,1-\alpha/2}}$. Where $x = \theta_i n$, θ is the haplotype frequency, n is number of blood sample (sample size) and α is the required width of the CI (α=0.95 for 95% CIs).

Table S1.1: Summary of the statistical methods for haplotype reconstruction.

	**MHF**	**R-EM**	**Bayesian**	**EM**	**MCMC**	**Gibbs**
Platform	DOS or windows	R code	R code	R code	R code	R code
Data that require (inputs)	Genotypes and MOI	Genotypes and MOI	Genotypes and MOI	Genotypes and MOI	Genotypes and MOI	Genotypes and MOI
Assumptions of each estimation	Observed MOI	Observed MOI	Incorporates prior with observed MOI	Observed MOI	Observed MOI	Observed MOI
Calculation method	Maximum likelihood hill climbing	Maximum likelihood EM-algorithm	MCMC Metropolis-Hastings	Maximum likelihood EM-algorithm	MCMC Metropolis-Hastings	MCMC Gibbs sampler
Estimates (outputs)	Haplotype probabilities and CI	Haplotype probabilities and SE	Haplotype probabilities and CI	Haplotype probabilities and CI	Haplotype probabilities and CI	Haplotype probabilities and CI
Dealing with missing values	No	Yes	Yes	Yes	Yes	Yes
Maximum number of SNPs	3 SNPs	No limit	7 SNPs	No limit	No limit	No limit
Runtime	Fast	Slow	Fast	Fast	Fast	Fast

MOI, Multiplicity of Infection; CI, confidence interval; SE, standard error.

Table S2: The correlation (R^2) of the estimated haplotype frequencies with simulated population and sample haplotype frequencies across statistical methods, and unknown MOI (no results can be obtained from MHF because that method cannot deal with unknown MOI). Higher value represents higher accuracy.

	R-EM	Bayesian	EM	MCMC	Gibbs
Population Haplotype (LoD_{SNP} / LoD_{MOI})					
0 / 0	0.754	0.947	0.977	0.970	0.962
0.10 / 0.05	0.848	0.957	0.978	0.971	0.964
0.20 / 0.10	0.920	0.959	0.976	0.970	0.967
0.30 / 0.15	0.954	0.956	0.973	0.967	0.968
Sample Haplotype (LoD_{SNP} / LoD_{MOI})					
0 / 0	0.770	0.955	0.983	0.975	0.968
0.10 / 0.05	0.864	0.963	0.984	0.977	0.971
0.20 / 0.10	0.934	0.965	0.983	0.977	0.974
0.30 / 0.15	0.966	0.963	0.981	0.975	0.976

LoD, Limit of Detection; SNP, Single Nucleotide Polymorphisms; MOI, Multiplicity of Infection.

<u>Table S3:</u> The similarity index (*IF*) of the estimated haplotype frequencies with simulated population and sample haplotype frequencies across statistical methods, and unknown MOI (no results can be obtained from MHF because that method cannot deal with unknown MOI). Higher value represents higher accuracy.

	R-EM	Bayesian	EM	MCMC	Gibbs
Population Haplotype (LoD_{SNP} / LoD_{MOI})					
0 / 0	0.793	0.904	0.938	0.928	0.926
0.10 / 0.05	0.841	0.912	0.943	0.933	0.929
0.20 / 0.10	0.884	0.909	0.938	0.930	0.928
0.30 / 0.15	0.910	0.898	0.926	0.919	0.921
Sample Haplotype (LoD_{SNP} / LoD_{MOI})					
0 / 0	0.801	0.911	0.944	0.932	0.931
0.10 / 0.05	0.850	0.918	0.949	0.939	0.935
0.20 / 0.10	0.895	0.917	0.946	0.937	0.936
0.30 / 0.15	0.923	0.906	0.935	0.926	0.930

LoD, Limit of Detection; SNP, Single Nucleotide Polymorphisms; MOI, Multiplicity of Infection.

<u>Table S4:</u> The *MSE* of the estimated haplotype frequencies with simulated population and sample haplotype frequencies across statistical methods, and unknown MOI (no results can be obtained from MHF because that method cannot deal with unknown MOI). Lower value represents higher accuracy.

	R-EM	Bayesian	EM	MCMC	Gibbs
Population Haplotype (LoD_{SNP} / LoD_{MOI})					
0 / 0	0.507	0.106	0.048	0.064	0.080
0.10 / 0.05	0.304	0.092	0.044	0.058	0.072
0.20 / 0.10	0.160	0.108	0.055	0.068	0.070
0.30 / 0.15	0.095	0.149	0.079	0.096	0.082
Sample Haplotype (LoD_{SNP} / LoD_{MOI})					
0 / 0	0.476	0.091	0.039	0.055	0.069
0.10 / 0.05	0.275	0.079	0.033	0.046	0.059
0.20 / 0.10	0.134	0.091	0.039	0.053	0.056
0.30 / 0.15	0.070	0.130	0.060	0.077	0.062

LoD, Limit of Detection; SNP, Single Nucleotide Polymorphisms; MOI, Multiplicity of Infection.

<u>Table S5:</u> The average change coefficient (*C*) of the estimated haplotype frequencies with simulated population and sample haplotype frequencies for haplotype frequency >5% across statistical methods and unknown MOI (no results can be obtained from MHF because that method cannot deal with unknown MOI). Lower value represents higher accuracy.

	R-EM	Bayesian	EM	MCMC	Gibbs
Population Haplotype (LoD_{SNP} / LoD_{MOI})					
0 / 0	39.3	19.8	13.0	15.5	15.4
0.10 / 0.05	31.8	18.1	12.5	14.4	15.1
0.20 / 0.10	24.6	18.6	13.3	14.9	15.4
0.30 / 0.15	19.5	20.2	15.2	16.8	16.6
Sample Haplotype (LoD_{SNP} / LoD_{MOI})					
0 / 0	38.3	18.3	11.8	14.4	14.3
0.10 / 0.05	30.3	16.9	11.0	13.1	13.9
0.20 / 0.10	22.6	17.0	11.8	13.7	14.0
0.30 / 0.15	17.0	18.9	13.6	15.5	15.0

LoD, Limit of Detection; SNP, Single Nucleotide Polymorphisms; MOI, Multiplicity of Infection.

Table S6: Percentages of simulated sample haplotype frequencies and population haplotype frequencies that fall outside the confidence intervals of the estimated haplotype frequencies across statistical methods and unknown MOI (no results can be obtained from MHF because that method cannot deal with unknown MOI). Lower value represents higher accuracy.

	R-EM	Bayesian	EM	MCMC	Gibbs
Population Haplotype (LoD_{SNP} / LoD_{MOI})					
0 / 0	29.3	17.4	0.7	1.6	2.4
0.10 / 0.05	19.8	15.2	1.1	1.6	2.2
0.20 / 0.10	11.8	17.4	2.1	2.7	2.9
0.30 / 0.15	9.6	23.2	4.6	5.5	4.7
Sample Haplotype (LoD_{SNP} / LoD_{MOI})					
0 / 0	30.8	17.2	4.4	5.5	5.6
0.10 / 0.05	20.6	15.5	4.3	4.6	5
0.20 / 0.10	11.7	16.9	4.4	4.7	5
0.30 / 0.15	8.9	22.2	5.6	6.3	5.8

LoD, Limit of Detection; SNP, Single Nucleotide Polymorphisms; MOI, Multiplicity of Infection.

<u>Table S7:</u> The correlation (R^2) of the estimated haplotype frequencies with simulated population and sample haplotype frequencies across statistical methods among 2 SNPs. Higher value represents higher accuracy.

	MHF	R-EM	Bayesian	EM	MCMC	Gibbs
Population Haplotype (LoD_{SNP} / LoD_{MOI})						
0 / 0	0.975	0.946	0.971	0.982	0.979	0.961
0.10 / 0.05	0.978	0.959	0.974	0.982	0.984	0.972
0.20 / 0.10	0.977	0.970	0.974	0.981	0.985	0.979
0.30 / 0.15	0.974	0.977	0.970	0.979	0.982	0.984
Sample Haplotype (LoD_{SNP} / LoD_{MOI})						
0 / 0	0.983	0.955	0.977	0.989	0.985	0.967
0.10 / 0.05	0.986	0.968	0.981	0.989	0.991	0.978
0.20 / 0.10	0.985	0.978	0.980	0.988	0.992	0.986
0.30 / 0.15	0.981	0.985	0.977	0.987	0.989	0.991

LoD, Limit of Detection; SNP, Single Nucleotide Polymorphisms; MOI, Multiplicity of Infection.

<u>Table S8:</u> The similarity index (*IF*) of the estimated haplotype frequencies with simulated population and sample haplotype frequencies across statistical methods among 2 SNPs. Higher value represents higher accuracy.

	MHF	R-EM	Bayesian	EM	MCMC	Gibbs
Population Haplotype (LoD_{SNP} / LoD_{MOI})						
0 / 0	0.949	0.918	0.943	0.957	0.941	0.925
0.10 / 0.05	0.949	0.932	0.945	0.958	0.957	0.942
0.20 / 0.10	0.939	0.944	0.938	0.953	0.960	0.954
0.30 / 0.15	0.922	0.949	0.924	0.944	0.946	0.956
Sample Haplotype (LoD_{SNP} / LoD_{MOI})						
0 / 0	0.959	0.923	0.950	0.965	0.945	0.926
0.10 / 0.05	0.959	0.938	0.953	0.967	0.965	0.945
0.20 / 0.10	0.947	0.952	0.945	0.961	0.969	0.961
0.30 / 0.15	0.929	0.959	0.930	0.952	0.953	0.966

LoD, Limit of Detection; SNP, Single Nucleotide Polymorphisms; MOI, Multiplicity of Infection.

<u>Table S9:</u> The *MSE* of the estimated haplotype frequencies with simulated population and sample haplotype frequencies across statistical methods among 2 SNPs. Lower value represents higher accuracy.

	MHF	R-EM	Bayesian	EM	MCMC	Gibbs
Population Haplotype (LoD_{SNP} / LoD_{MOI})						
0 / 0	0.115	0.278	0.134	0.083	0.141	0.254
0.10 / 0.05	0.114	0.196	0.130	0.082	0.078	0.158
0.20 / 0.10	0.162	0.138	0.170	0.104	0.072	0.098
0.30 / 0.15	0.254	0.115	0.253	0.142	0.132	0.083
Sample Haplotype (LoD_{SNP} / LoD_{MOI})						
0 / 0	0.076	0.246	0.104	0.054	0.119	0.236
0.10 / 0.05	0.073	0.162	0.096	0.050	0.051	0.136
0.20 / 0.10	0.120	0.102	0.133	0.068	0.041	0.070
0.30 / 0.15	0.209	0.076	0.213	0.103	0.095	0.050

LoD, Limit of Detection; SNP, Single Nucleotide Polymorphisms; MOI, Multiplicity of Infection.

Table S10: The average change coefficient (*C*) of the estimated haplotype frequencies with simulated population and sample haplotype frequencies for haplotype frequency >5% across statistical methods among 2 SNPs. Lower value represents higher accuracy.

	MHF	R-EM	Bayesian	EM	MCMC	Gibbs
Population Haplotype (LoD_{SNP} / LoD_{MOI})						
0 / 0	13.6	20.0	14.0	10.7	14.9	16.8
0.10 / 0.05	13.6	16.8	13.7	10.6	11.2	13.2
0.20 / 0.10	15.8	14.4	15.7	11.7	10.2	11.2
0.30 / 0.15	19.6	13.5	18.7	14.0	13.6	11.8
Sample Haplotype (LoD_{SNP} / LoD_{MOI})						
0 / 0	10.9	19.0	12.4	9.1	14.4	16.6
0.10 / 0.05	11.0	15.4	11.7	8.4	9.7	12.3
0.20 / 0.10	13.7	12.4	13.8	9.6	8.2	9.3
0.30 / 0.15	18.1	11.0	17.3	12.1	11.8	9.5

LoD, Limit of Detection; SNP, Single Nucleotide Polymorphisms; MOI, Multiplicity of Infection.

Table S11: Percentages of simulated sample haplotype frequencies and population haplotype frequencies that fall outside the confidence intervals of the estimated haplotype frequencies across statistical methods among 2 SNPs. Lower value represents higher accuracy.

	MHF	R-EM	Bayesian	EM	MCMC	Gibbs
Population Haplotype (LoD_{SNP} / LoD_{MOI})						
0 / 0	4.5	6.7	13.4	0.7	4.3	10.1
0.10 / 0.05	6.8	5.4	14.1	0.8	0.9	4.4
0.20 / 0.10	16.4	5	21.2	1.3	0.6	1.8
0.30 / 0.15	30.8	7.6	30.5	3.6	3.1	1.4
Sample Haplotype (LoD_{SNP} / LoD_{MOI})						
0 / 0	2.6	5.9	9.8	1	3.7	9.9
0.10 / 0.05	3.3	4.2	10.3	0.9	1.3	3.6
0.20 / 0.10	11.4	3.6	16	1.1	1	1.2
0.30 / 0.15	27.6	5.7	27.1	2	1.8	1.3

LoD, Limit of Detection; SNP, Single Nucleotide Polymorphisms; MOI, Multiplicity of Infection.

Figure S1: A trace plot and density plots of the iteration number against the value of the haplotype frequencies at 500 iterations.

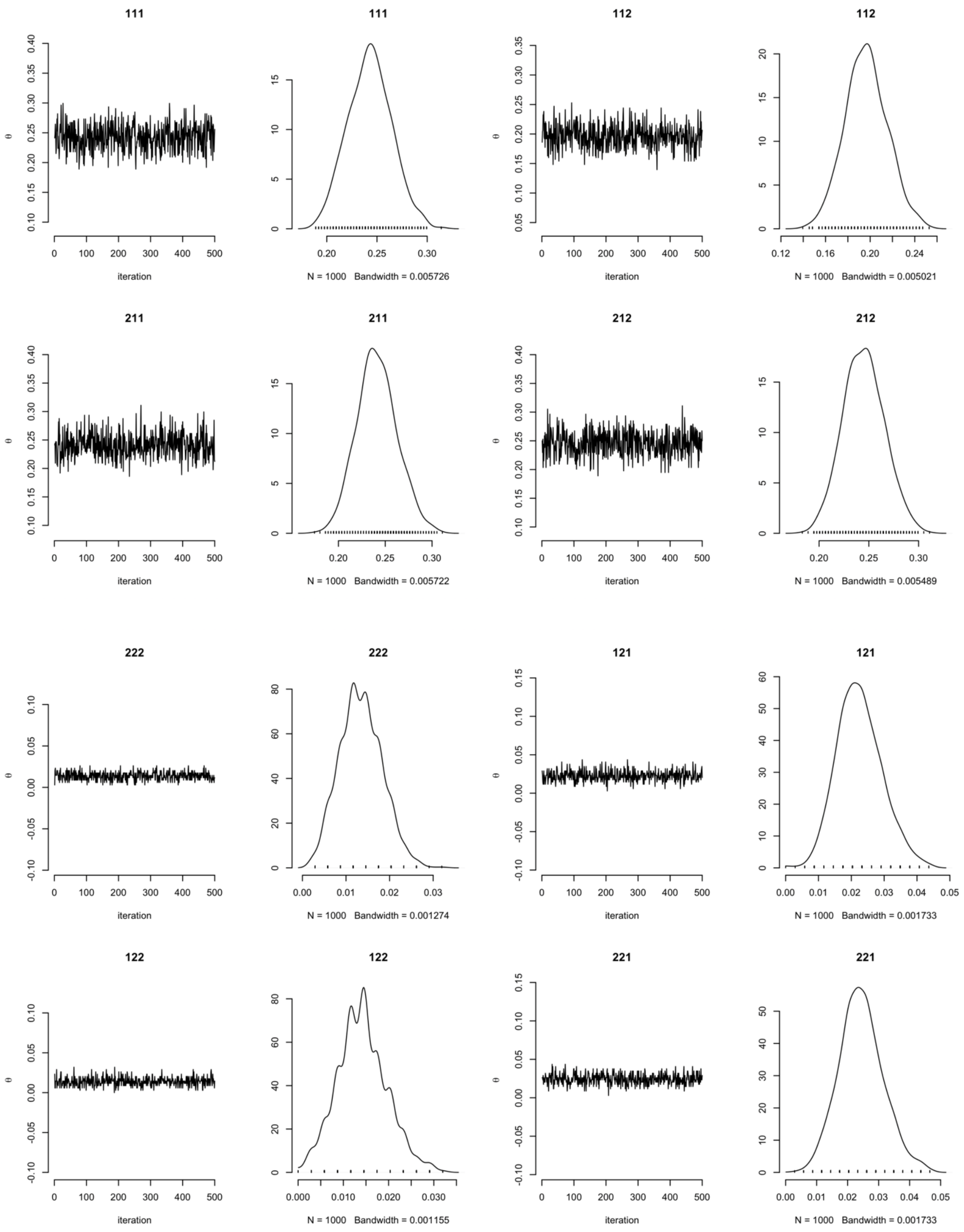

Figure S2: Autocorrelation plots to assess the autocorrelations between the haplotype frequencies of Markov chain at 500 iterations.

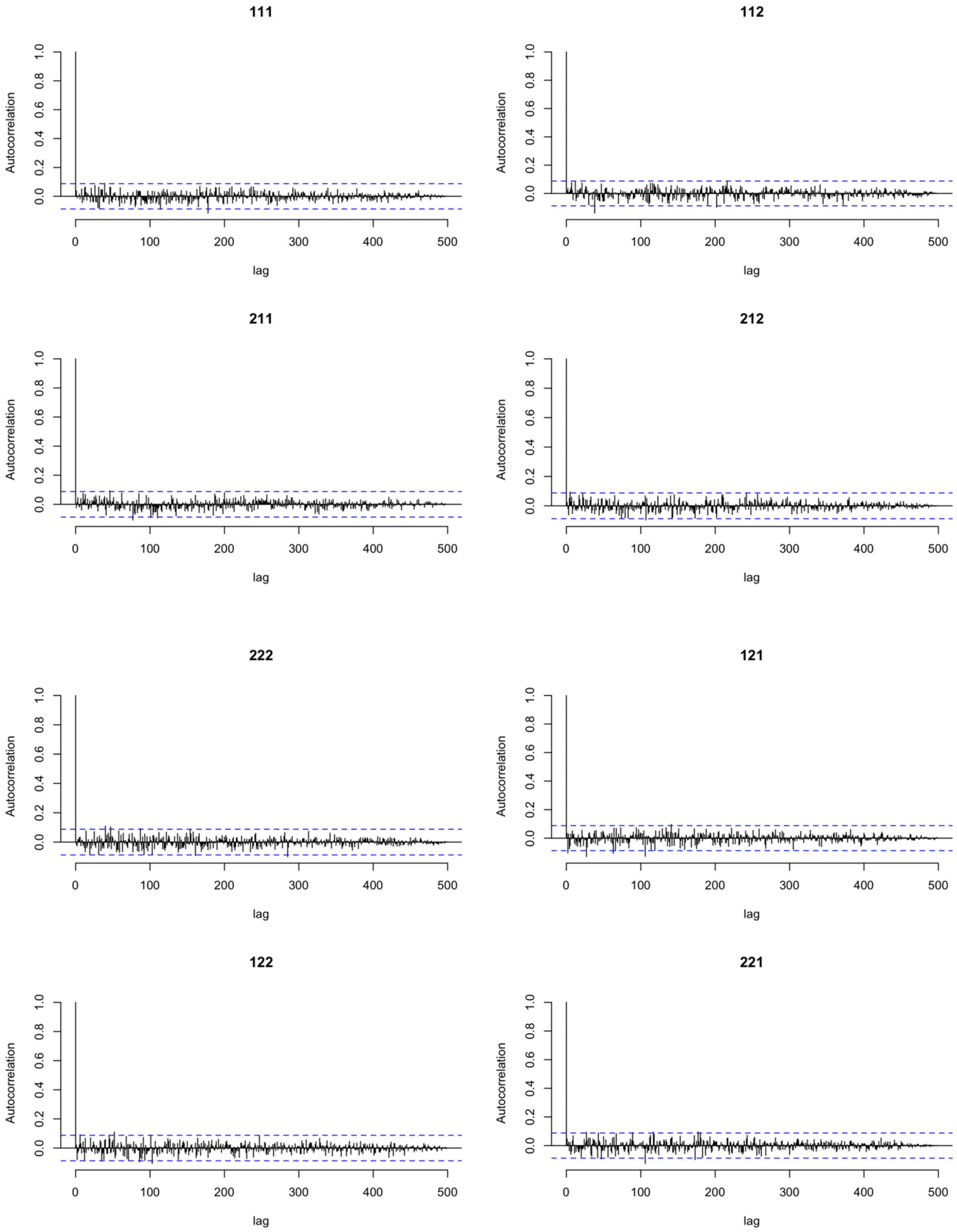

Figure S3: Gelman Rubin Brooks plot (shrink factor as the number of iterations) and mean plot (mean as the number of iterations) with 2 chains at 500 iterations.

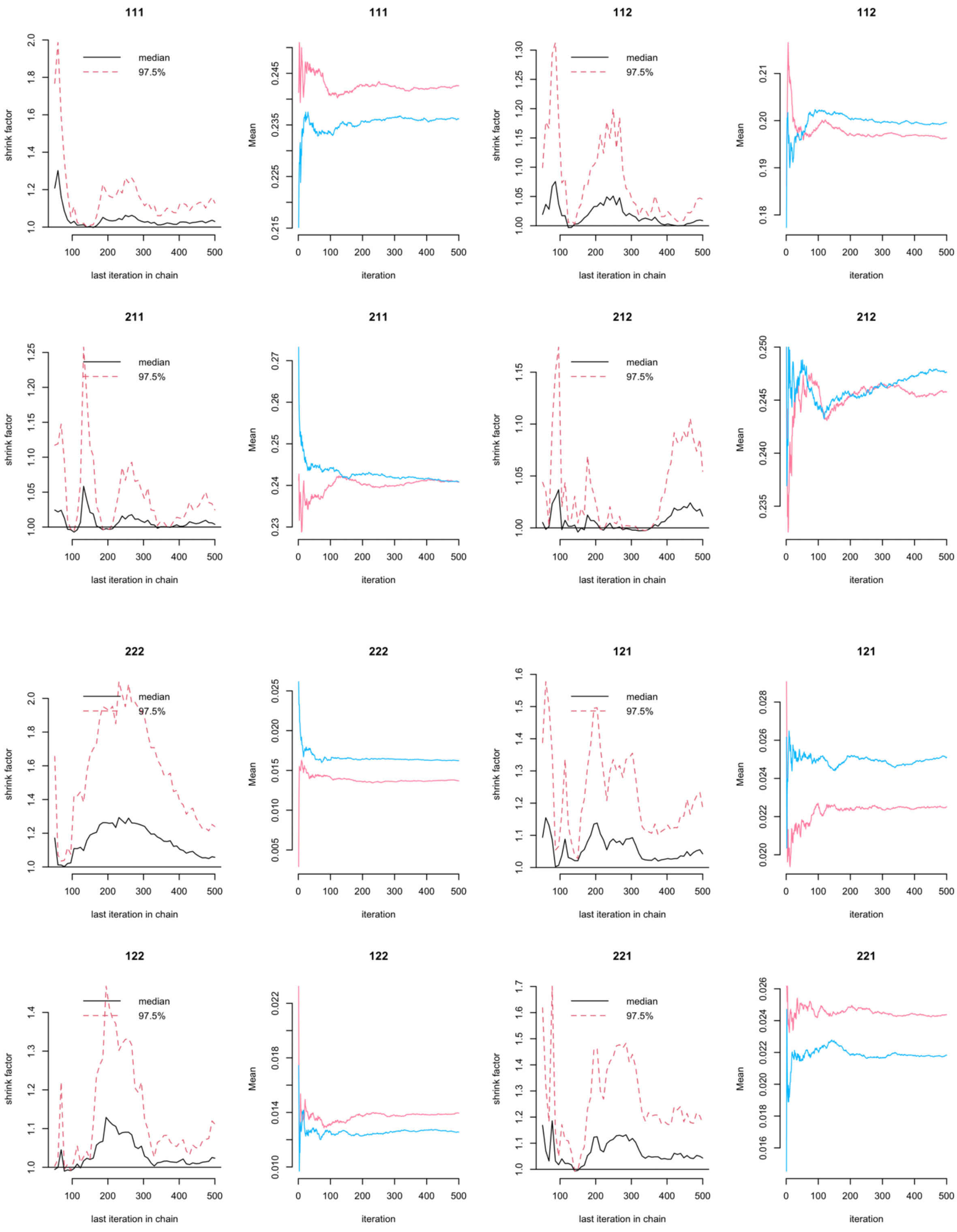

Figure S4: A trace plot and density plots of the iteration number against the value of the haplotype frequencies at 1000 iterations.

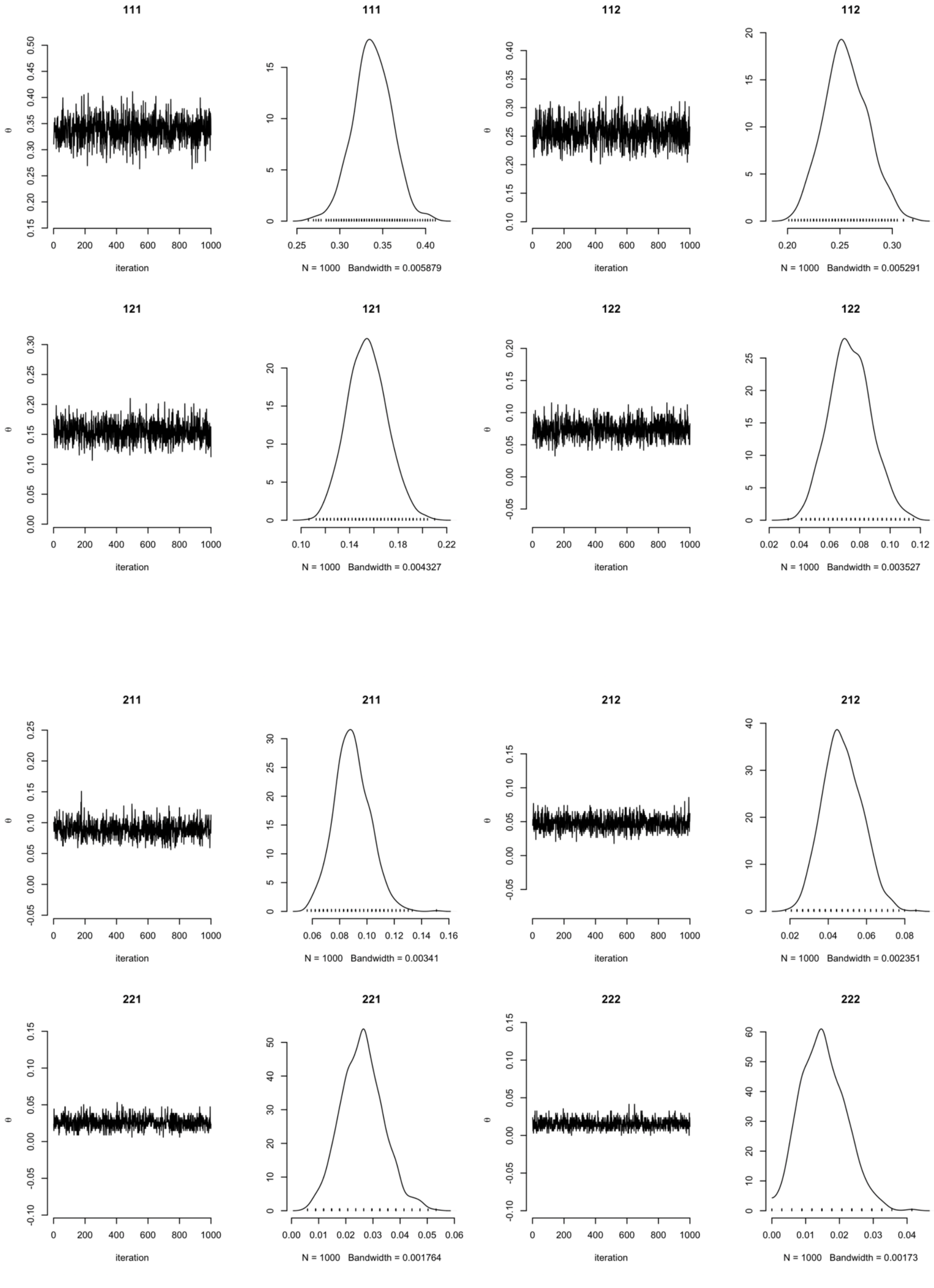

Figure S5: Autocorrelation plots to assess the autocorrelations between the haplotype frequencies of Markov chain at 1000 iterations.

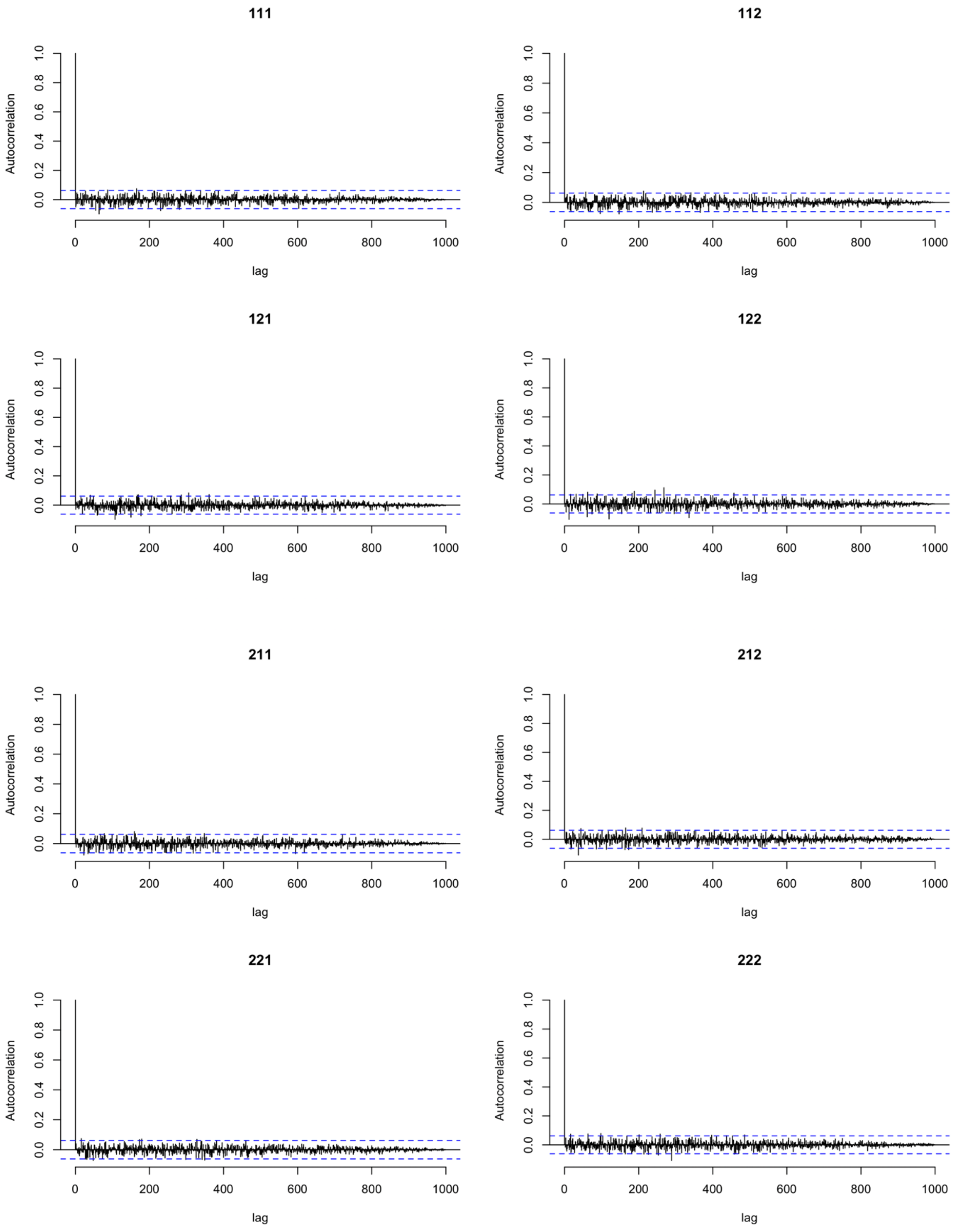

Figure S6: Gelman Rubin Brooks plot (shrink factor as the number of iterations) and mean plot (mean as the number of iterations) with 2 chains at 1000 iterations.

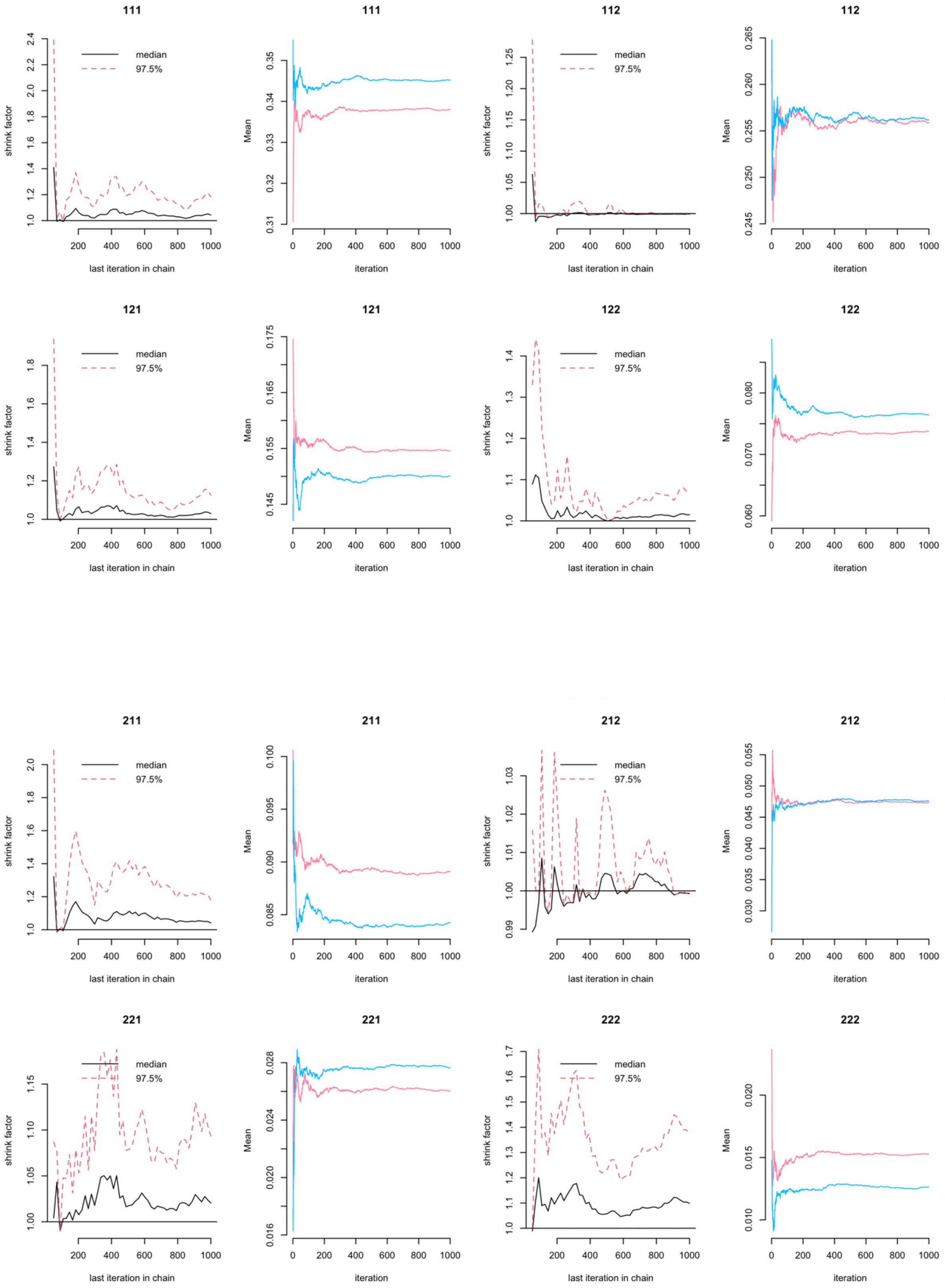

Supplement: Supplementary file 1 — Additional file 1: Includes data that support and expand some of the interpretations and conclusions drawn in the main text. [file 12936_2021_3841_MOESM1_ESM.pdf]
